# Effects of Human Recombinant Growth Hormone (rhGH) Treatment on Plasma Extracellular Vesicles in GH-Deficient Children: A Preliminary Report

**DOI:** 10.3390/jcm15072528

**Published:** 2026-03-26

**Authors:** Antonello E. Rigamonti, Luca Ferrari, Chiara Favero, Mirjam Hoxha, Adele Bondesan, Nicoletta Marazzi, Silvano G. Cella, Alessandro Sartorio

**Affiliations:** 1 Department of Clinical Sciences and Community Health, Dipartimento di Eccellenza 2023–2027, University of Milan, 20129 Milan, Italy; silvano.cella@unimi.it; 2 EPIGET Laboratory, Department of Clinical Sciences and Community Health, Dipartimento di Eccellenza 2023–2027, University of Milan, 20122 Milan, Italy; luca.ferrari@unimi.it (L.F.); chiara.favero@unimi.it (C.F.); mirjam.hoxha@unimi.it (M.H.); 3 Occupational Health Unit, Fondazione IRCCS Ca’ Granda Ospedale Maggiore Policlinico, 20122 Milan, Italy; 4 Experimental Laboratory for Auxo-Endocrinological Research, Istituto Auxologico Italiano, IRCCS (Istituto di Ricovero e Cura a Carattere Scientifico), 28824 Piancavallo-Verbania, Italy; a.bondesan@auxologico.it (A.B.); n.marazzi@auxologico.it (N.M.); sartorio@auxologico.it (A.S.)

**Keywords:** GH deficiency, children, extracellular vesicles, recombinant human GH, IGF-1, osteocalcin, adipose tissue, immune system

## Abstract

**Background**: Recombinant human growth hormone (rhGH) replacement therapy, administered to children with growth hormone deficiency (GHD), exerts pleiotropic effects on growth, metabolism, and tissue functions. Extracellular vesicles (EVs) are emerging mediators of inter-organ communication, but the effects of rhGH therapy on EV release in humans have not yet been investigated. **Methods**: In a preliminary prospective clinical study, children with GHD (n = 10; F/M = 5/5; age: 11.0 ± 2.7 years) were treated with rhGH for 6 months. Plasma samples were collected at baseline (T0) and after treatment (T6) to characterize the size distribution and tissue-derived composition of circulating EVs. Total EVs and EV subpopulations derived from monocytes/macrophages (CD14+), adipose tissue (FABP+), skeletal muscle (SCG+), endothelium (CD62E+), and platelets (CD42A+) were analyzed. Clinical, auxological/auxometric, and biochemical/metabolic parameters were assessed in parallel. Statistical methods included longitudinal analyses, interaction models, and adjustments for relevant covariates, including insulin-like growth factor 1 (IGF-1) and osteocalcin. **Results**: After 6 months of rhGH therapy, significant improvements in height velocity (cm/year and SDS) were observed, accompanied by increased circulating IGF-1 and osteocalcin levels. Hormone therapy induced no size-dependent changes in (total) EVs. Significant increases in CD14+ and FABP+ EVs were observed after treatment, without affecting the other tissue-derived EVs. Interaction analyses revealed that children with more severe GHD exhibited a stronger vesiculogenic response to rhGH. Furthermore, specific tissue-derived EVs were associated with metabolic/biochemical and auxological/auxometric parameters, including lipids, insulin resistance, and growth-related measures. **Conclusions**: When administered for six months, rhGH therapy seems to selectively change tissue-derived composition of circulating EVs in GHD children, particularly those derived from immune cells and adipose tissue. These preliminary findings suggest that EVs might represent an adjunctive component of GH-dependent inter-organ communication and might serve as biomarkers of treatment response and disease severity in pediatric endocrinology.

## 1. Introduction

It is well established that, in pediatric age, growth hormone (GH) not only regulates statural growth, but also exerts multisystemic effects on muscle trophism, cardiovascular function, metabolic status, psychological development, and overall quality of life, thus further supporting the need for hormone replacement therapy in subjects with GH deficiency (GHD) [1,2,3].

Unfortunately, beyond the binding of GH to its receptor, which leads to the production of insulin-like growth factor 1 (IGF-1) in the liver and other tissues, the cellular and molecular mechanisms underlying the GH-mediated interaction among various, even distant, tissues, including muscle, bone, adipose tissue, immune system, and heart, are not fully understood to date [4,5].

The extracellular *milieu*, primarily the bloodstream, is not hospitable to numerous GH-dependent biomolecules, which are extremely labile and readily inactivated by plasma proteases and RNases. During the evolution of unicellular and multicellular organisms, a sophisticated vesicle-mediated transport system has emerged, represented by extracellular vesicles (EVs) [6,7]. EVs are lipid bilayer–enclosed particles that carry proteins, nucleic acids (including mitochondrial-derived nucleic acids), and metabolites, and are involved in a wide range of physiological and pathological processes [8,9].

EVs mediate the intercellular transfer of diverse biomolecules between cells and across tissues [10]. Circulating EV levels are modulated by endocrine stimuli, including GH, and originate from multiple GH-responsive tissues [11]. However, apart from a limited number of in vitro studies in tumour cell models, the effects of GH on EV size distribution and tissue origin in humans—such as in subjects with GHD—remain largely unknown [12].

The systemic benefits of GH, such as the stimulation of statural growth and musculoskeletal trophism, improvement of gluco-metabolic control, reduction in cardiovascular risk, and redistribution/transformation of white/brown adipose tissue, are thought to be mediated by EVs, which, under the action of GH and/or IGF-1, can function in an autocrine, paracrine, and endocrine manner, transporting GH-dependent biomolecules within and over the cytomembrane [13,14].

Therefore, based on these premises, studying plasmatic EV release in children with GHD undergoing recombinant human GH (rhGH) therapy could provide a promising tool to biochemically monitor the multisystemic efficacy of this treatment, complementing auxometric evaluation and determination of circulating IGF-1 levels, which are traditionally performed during follow-up visits of children with short stature due to GHD [15].

The primary objective of the present study was to evaluate the size and tissue-derivational profiles of EVs generated in children with GHD undergoing rhGH replacement therapy. Secondary objectives were to correlate EV concentrations with auxometric and biochemical parameters used in clinical-endocrine practice to evaluate short stature.

The results of the present study could provide preliminary information to more rationally set up clinical and biochemical follow-up of rhGH replacement therapy, as well as to elucidate the molecular and cellular mechanisms underlying the multi-systemic action of GH, the most important anabolic hormone in humans [16].

## 2. Materials and Methods

### 2.1. Study Population and Experimental Design

A total of 10 pediatric patients, both genders (5 females and 5 males), were enrolled at the Research Centre for Growth Disorders, Istituto Auxologico Italiano, IRCCS, Milan, Italy. All participants were diagnosed with isolated GHD in accordance with the criteria defined by the Italian Medicines Agency (AIFA), note 39. Specifically, inclusion criteria comprised short stature (height ≤ −3 SD or ≤ −2 SD accompanied by an annual height velocity ≤ −1.0 SD for age and sex, assessed over a minimum period of six months) and a GH peak concentration < 8 ng/mL during two distinct pharmacological stimulation tests.

Children presenting with structural abnormalities of the hypothalamic–pituitary region, as assessed by cerebral magnetic resonance imaging (MRI), were excluded from both the study and rhGH treatment.

At baseline (T0, before treatment), demographic information and clinical, anthropometric, and auxological parameters were collected for all subjects. Body composition was assessed using bioelectrical impedance analysis. The same evaluations were repeated after six months of rhGH therapy (T6).

All patients received daily subcutaneous rhGH at a dose of 0.025–0.035 mg/kg body weight, corresponding to approximately 0.7–1.0 mg/m^2^ body surface area.

After an overnight fast of at least 12 h, venous blood samples were obtained at T0 (before treatment) and T6 (6 months after therapy), approximately 10–12 h after the last rhGH administration.

The study protocol was reviewed and approved by the Ethics Committee (EC) of the Istituto Auxologico Italiano, IRCCS, Milan, Italy (EC code: CE: 2022_03_15_02; date of EC approval: 15 March 2022; research project code: 01C211; acronym: VESCIGHTP).

### 2.2. Metabolic and Biochemical Assessments

Serum or plasma concentrations of total cholesterol (T-C), high-density lipoprotein cholesterol (HDL-C), low-density lipoprotein cholesterol (LDL-C), triglycerides (TG), glucose, insulin, IGF-1, osteocalcin, and high-sensitivity C-reactive protein (hsCRP) were measured at T0 and T6.

Serum lipid parameters (T-C, LDL-C, HDL-C, and TG) were quantified using enzymatic colorimetric assays (Roche Diagnostics, Monza, Italy). The analytical sensitivities were 3.86 mg/dL for T-C, 3.87 mg/dL for LDL-C, 3.09 mg/dL for HDL-C, and 8.85 mg/dL for TG.

Fasting serum glucose levels were determined using the glucose oxidase method (Roche Diagnostics, Monza, Italy), with a detection limit of 2 mg/dL. Serum insulin concentrations were measured using a chemiluminescent immunometric assay (Elecsys Insulin, Roche Diagnostics, Monza, Italy), with a sensitivity of 0.2 μIU/mL.

Hs-CRP levels were assessed by an immunoturbidimetric method (CRP RX, Roche Diagnostics GmbH, Mannheim, Germany), with a lower detection limit of 0.3 mg/L.

The intra- and inter-assay coefficients of variation were as follows: 1.1% and 1.6% for T-C; 1.2% and 2.5% for LDL-C; 1.8% and 2.2% for HDL-C; 1.1% and 2.0% for TG; 1.0% and 1.3% for glucose; and 1.5% and 4.9% for insulin.

Plasma IGF-1 concentrations were measured using an enzyme-labelled chemiluminescent immunometric assay (Mediagnost GmbH, Tübingen, Germany), with a sensitivity of 10 ng/mL. The intra- and inter-assay coefficients of variation were 3.5% and 7%, respectively.

Plasma osteocalcin levels were measured using a commercial electrochemiluminescence immunoassay (ECLIA) kit (Roche Diagnostics), with a sensitivity of 0.5 mcg/L.

Insulin resistance was estimated for each subject using the homeostasis model assessment of insulin resistance (HOMA-IR), calculated according to the formula:

HOMA-IR = (insulin [μIU/mL] × glucose [mmol/L])/22.5.

### 2.3. Characterization of Size Distribution and Cell-Origin of EVs

Blood was collected into EDTA-containing tubes and transported at +4 °C to the EPIGET Lab (University of Milan) from Istituto Auxologico Italiano within 3 h after blood sampling. The first processing step was performed at the EPIGET Lab, where samples were centrifuged at 1200× *g* for 15 min at room temperature to obtain platelet-free plasma. An aliquot of 1.6 mL of plasma was supplemented with 0.04 M sucrose, kept overnight at −80 °C in a cooling box, and then stored at −80 °C until use for EV isolation.

Count and size of EVs were assessed by NTA (nanoparticle tracking analysis), a technique that measures the Brownian motion of vesicles suspended in fluid and displays them in real time through a high-sensitivity CCD camera. Using a NanoSight NS300 system (Malvern Panalytical Ltd., Malvern, UK, EVs were detected by laser light scattering. Five 30-s recordings were made for each sample. Collected data were analyzed using NTA software v. 3.3 (Malvern Instruments Ltd., Worcestershire, UK)), which provides high-resolution particle-size distribution profiles and concentration measurements of the EVs (count/mL in plasma).

The cell origin of EVs was characterized by flow cytometry using Northern lights (Cytek) spectral cell analyzer. Shortly, Kisker Biotech silica particles plain fluo green (100, 200, and 500 nm) were used to set the calibration gate on the analyzer. To analyze EV integrity, 30 μL aliquots were stained with 2 μM 5(6)-carboxyfluorescein diacetate N-succinimidyl ester (CFSE) at 37 °C for 30 min in the dark. Each aliquot of CFSE-stained sample was incubated with a specific antibody: CD14-APC (clone TÜK4) for monocyte/macrophage-derived EVs, CD62E (clone REA280) for endothelium-derived EVs, CD42a-APC (clone REA209) for platelet-derived EVs (Miltenyi Biotec, Bergisch Gladbach, Germany), A-FABP (clone B-4) for adipocyte-derived EVs, and α-sarcoglycan SCGA (clone F-7) for skeletal muscle-derived EVs (Santa Cruz Biotechnology, Dallas, TX, USA). Before use, each antibody was centrifuged at 17,000× *g* for 30 min at 4 °C to eliminate aggregates. The stained PBS control sample was used to detect the antibody’s autofluorescence. Quantitative multiparameter analysis of flow cytometry data was performed using FlowJo Software (v. 10) (Tree Star, Inc., Morganville, NJ, USA).

For further details, see also ref. [17].

### 2.4. Statistical Analysis

Baseline and post-treatment demographic, biochemical, and clinical variables were compared using paired statistical tests. Continuous variables were assessed for normality and expressed as mean ± standard deviation when normally distributed or as median and interquartile range [Q1, Q3] otherwise. Paired Student’s *t* tests or Wilcoxon signed-rank tests were applied accordingly. Categorical variables were summarized as counts and percentages. Individual trajectories of total and tissue-derived EVs were visualized using *spaghetti* plots.

Since the aim of the study was to evaluate longitudinal changes in total and tissue-derived EVs in children with GHD before and after 6 months of rhGH treatment, linear mixed-effects regression models for paired data were applied. Total EVs and tissue-derived EV subpopulations (CD14+, CD62E+, SCG+, CD42A+, FABP+) were log-transformed (natural logarithm) to approximate normal distributions. A random intercept was assigned to each participant to account for within-subject correlation. Models included rhGH treatment (T0 vs. T6) as a fixed effect and were adjusted for circulating IGF-1 levels, the main downstream mediator of GH action. For FABP+ EVs, models were additionally adjusted for osteocalcin, given its endocrine and metabolic role and its significant treatment-related variation. Best model selection was guided by minimizing the Akaike information criterion and maximizing the model’s explained variance. Results were reported as marginal geometric means with 95% confidence intervals, obtained by back-transforming estimated marginal means from log-scale models.

To investigate size-dependent effects of rhGH treatment on EV concentrations, linear mixed-effect models for paired data were applied separately for each EV size (30–700 nm), adjusting for IGF-1. Marginal geometric means and 95% CI at T0 and T6 were estimated for each size. Due to the high number of comparisons across EV sizes, multiple testing was addressed using the Benjamini–Hochberg false discovery rate (FDR) procedure, and FDR-adjusted *p*-values were reported.

Potential effect modification of clinical indicators of GHD severity (including height velocity, height, and GH peak to pharmacological stimulation tests) on the association between rhGH treatment and EV concentrations was evaluated by introducing interaction terms between rhGH treatment and each modifier in mixed-effects models. Associations between EV subpopulations and biochemical or auxological parameters were examined using linear mixed-effects regression models with random subject intercepts. Effect modification by rhGH treatment was assessed by introducing interaction terms between EVs and treatment status. When significant interactions were detected, treatment-specific associations were reported. In the absence of interaction, models were refitted, including main effects only.

All statistical analyses and graphical representations were performed using SAS software (version 9.4; SAS Institute, Cary, NC, USA).

A two-sided *p*-value < 0.05 was considered statistically significant.

## 3. Results

### 3.1. Comparison of Parameters Before and After rhGH Treatment

Baseline and post-treatment demographic, biochemical, and clinical characteristics in children with GHD are summarized in Table 1.

For normal distributions, values are reported as mean ± standard deviation, and, when it is possible, we applied a paired *t*-test. When values are not normally distributed, they are expressed as the median [Q1, Q3], and we used the Wilcoxon signed-rank test for paired data. Categorical data are reported as frequencies and percentages.

After six months of rhGH therapy, significant increases were observed in height velocity (HV) and height velocity standard deviation score (HV SDS), calculated using age- and sex-specific Italian growth reference charts [18]. These auxological improvements were accompanied by a significant rise in circulating IGF-1 levels. In addition, metabolic parameters, including plasma glucose, insulin, and total cholesterol concentrations, as well as the HOMA-IR value, were significantly higher at the end of the treatment period than at baseline. Finally, circulating osteocalcin levels increased significantly after the six-month treatment period, confirming the treatment’s efficacy in promoting bone growth.

### 3.2. Effect of rhGH Treatment on Circulating EVs

When considering each EV size or size subsets, rhGH treatment was associated with no significant changes in total EVs, indicating that the hormone therapy did not induce a uniform or specific increase/decrease in EV size composition (Figure S1 in the Supplementary Material).

**Figure 1:**
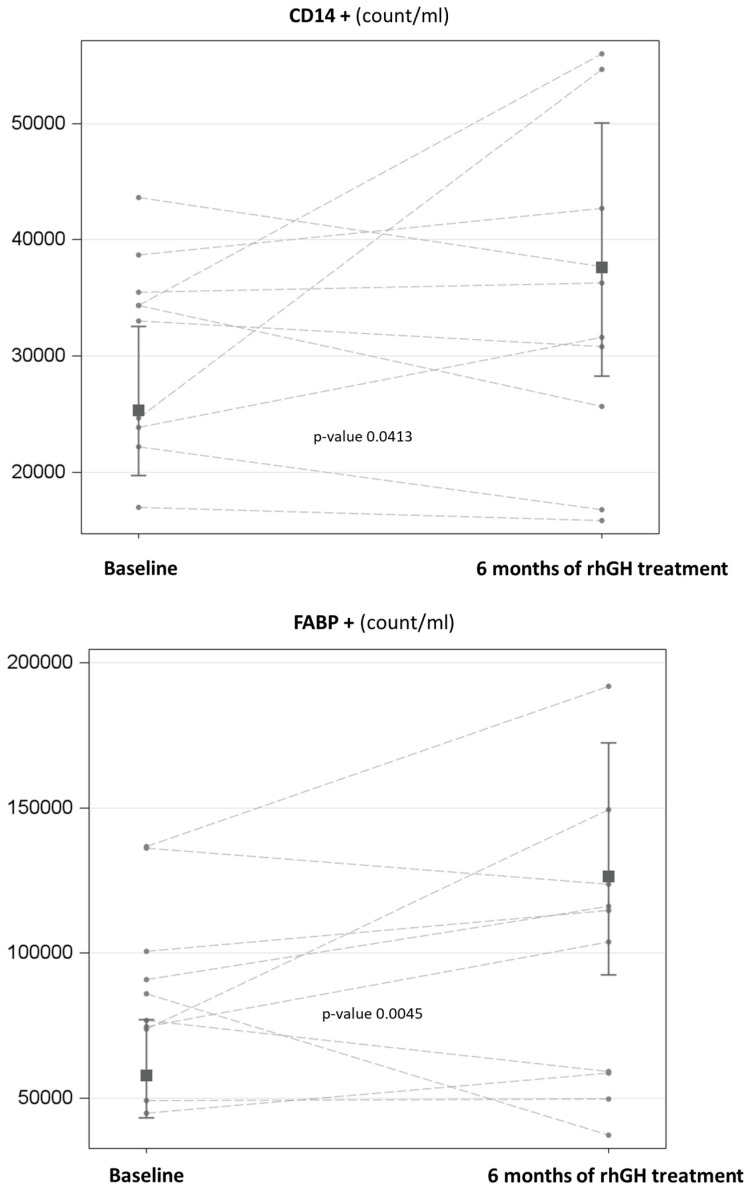
(**Top panel**): Geometric marginal means of CD14+ by time (i.e., baseline vs. 6 months of rhGH treatment), adjusted for IGF-1 levels, with the 95% confidence interval and the *p*-value derived from the comparison. (**Bottom panel**): Geometric marginal means of FABP+ by time (i.e., baseline vs. 6 months of rhGH treatment), adjusted for IGF-1 and osteocalcin levels, with the 95% confidence interval and the *p*-value derived from the comparison.

When assessing the impact of rhGH treatment on the tissue-derived composition of circulating EVs, comparisons between T6 and T0 revealed significant time-dependent increases in CD14+ EVs (∆% = 1.49; 95% CI = 1.44–1.54; *p*-value = 0.0431) and FABP+ EVs (∆% = 2.18; 95% CI = 2.13–2.23; *p*-value = 0.0045), without affecting total EVs or other specific tissue-derived EVs (Table 2; Figure 1).

Inter-individual variability in EV concentrations in response to rhGH treatment was evident in *spaghetti* plot representations of (total and tissue-derived) EVs. As shown in Figure S2 in the Supplementary Material, total and specific tissue-derived EVs varied, highlighting that the magnitude of change between T0 and T6 differed across participants, with some showing a greater response to the hormone therapy.

To investigate potential determinants of this variability, we assessed whether clinical and biochemical factors—such as height SDS, HV (expressed in cm/year or SDS), and GH peak to pharmacological tests (clonidine and arginine)—modified the effect of rhGH on EVs (Tables S1–S5 in the Supplementary Material). Among these factors, only HV SDS was identified as a significant effect modifier on rhGH-induced change in CD62E+ EVs (−4 SDS: ∆% = 2.49; 95% CI = [3.27; 1.89]; −3 SDS: (∆% = 1.63; 95% CI = [2.04; 1.31]; −2 SDS: (∆% = 1.07; 95% CI = [1.50; 0.76]; −1 SDS: (∆% = 0.70; 95% CI = [1.17; 0.42]; *p*-value of interaction = 0.0235), suggesting that children with more severe GHD (i.e., having lower HV SDS) showed an increased CD62E+ response to rhGH over time.

### 3.3. Associations Between EVs and Biochemical/Clinical Parameters

Results of association analyses between (total and specific tissue-derived) EVs and biochemical/clinical parameters are summarized in Tables S6 and S7 in the Supplementary Material.

Shortly, rhGH treatment was identified as a significant effect modifier on the following associations: CD62E+ EVs with TC (T0: ∆% = 1.81; 95% CI = [−0.17; 3.83]; T6: ∆% = −0.68; 95% CI = [−1.96; 0.62]; *p*-value of interaction = 0.0209) and LDL-C (T0: ∆% = 2.06; 95% CI = [−0.77; 4.97]; T6: ∆% = −0.83; 95% CI = [−2.09; 0.45]; *p*-value of interaction = 0.0470); CD14+ EVs with weight (kg) (T0: ∆% = 0.16; 95% CI = [−1.85; 2.21]; T6: ∆% = −1.76; 95% CI = [−3.96; 0.48]; *p*-value of interaction = 0.0203), weight (SDS) (T0: ∆% = 9.64; 95% CI = [−13.07; 38.28]; T6: ∆% = −24.64; 95% CI = [−67.88; 76.83]; *p*-value of interaction = 0.0222), BMI (kg/m^2^) (T0: ∆% = 1.18; 95% CI = [−4.58; 7.29]; T6: ∆% = −5.21; 95% CI = [−12.01; 2.11]; *p*-value of interaction = 0.0182), BMI (SDS) (T0: ∆% = 8.32; 95% CI = [−11.28; 32.24]; T6: ∆% = −15.02; 95% CI = [−34.90; 10.94]; *p*-value of interaction = 0.0166); and TG (T0: ∆% = −0.02; 95% CI = [−1.26; 1.24]; T6: ∆% = −1.49; 95% CI = [0.74; 2.24]; *p*-value of interaction = 0.0350); CD42A+ EVs with HV SDS (T0: ∆% = 16.03; 95% CI = [−5.06; 41.80]; T6: ∆% = −11.82; 95% CI = [−21.80; −0.56]; *p*-value of interaction = 0.0242); FABP+ EVs with HV (cm/year) (T0: ∆% = 6.78; 95% CI = [−5.51; 20.66]; T6: ∆% = −12.16; 95% CI = [−19.35; −4.33]; *p*-value of interaction = 0.0152) and HV (SDS) (T0: ∆% = 11.63; 95% CI = [−0.72; 25.51]; T6: ∆% = −15.77; 95% CI = [−25.38; −4.91]; *p*-value of interaction = 0.0044).

After adjusting for rhGH treatment, the following associations were significant: SGC+ EVs with HbA1c (∆% = −94.88; 95% CI = [−99.21; −66.75]; *p*-value = 0.0057); CD42A+ with height SDS (∆% = −76.61; 95% CI = [−90.29; −43.67]; *p*-value = 0.0041); FABP+ EVs with height (cm) (∆% = −2.00; 95% CI = [−3.50; −0.48]; *p*-value = 0.0164), insulin (∆% = −6.81; 95% CI = [−11.23; −2.17]; *p*-value = 0.0075), HOMA-IR (∆% = −23.80; 95% CI = [−39.82; −3.53]; *p*-value = 0.0272), and osteocalcin (∆% = −0.90; 95% CI = [−1.48; −0.32]; *p*-value = 0.0059).

## 4. Discussion

### 4.1. Impact of rhGH on the EV Concentration

The present preliminary study shows that rhGH therapy can alter the plasma EV tissue-derived composition—rather than size distribution—in children with GHD, supporting the concept that EV release can be influenced by endocrine signals (such as pituitary hormones) in humans [19].

While EVs are well recognized as mediators of intercellular communication in metabolic and inflammatory conditions via endocrine/paracrine/autocrine mechanisms, their regulation by pituitary hormones has remained largely unexplored in vivo [20].

A key finding of our preliminary study is the selective increase in monocyte/macrophage-derived (CD14+) and adipose-tissue-derived (FABP+) EVs following six months of rhGH therapy, whereas total EVs and those derived from skeletal muscle, endothelium, and platelets were not significantly affected (SCG+, CD42A+ and CD62E+, respectively).

These findings suggest that rhGH does not globally stimulate EV release but instead is associated with changes in specific circulating EV subpopulations, reflecting a tissue-dependent response consistent with the heterogeneous regulation of EV release across different cell types [21].

### 4.2. GH-Dependent Modulation of Immune- and Adipose-Derived EVs

The increase in CD14+ EVs suggests that cells of the monocyte–macrophage lineage are particularly sensitive to GH stimulation and its vesiculogenic signalling. Indeed, monocytes and macrophages express functional GH receptors, and GH has been shown to modulate their metabolic activity, inflammatory phenotype, and intracellular signalling pathways, all of which are known regulators of EV biogenesis/release [22,23].

Immune-derived EVs have been implicated in metabolic regulation and endocrine–immune crosstalk, further supporting the biological relevance of the observed increase in CD14+ EVs during rhGH treatment [24].

Similarly, as demonstrated in the present study, the increase in FABP+ EVs seems to indicate that adipose tissue is a major contributor to GH-induced changes in circulating EVs. Adipose tissue is a primary metabolic and endocrine target of GH, which promotes lipolysis, alters adipocyte differentiation, and modulates adipose tissue inflammation, processes tightly linked to EV secretion [25]. Moreover, adipocyte-derived EVs are increasingly recognized as endocrine mediators that influence insulin sensitivity, lipid metabolism, and inflammatory signalling in distant tissues [26].

Therefore, the increase in FABP+ EVs observed in our cohort of GHD children might reflect a physiological adaptation of adipose tissue to a GH-driven metabolic response.

### 4.3. Lack of Effect on Skeletal Muscle, Endothelial, and Platelet EVs

In contrast to the previously described significant findings, rhGH treatment did not significantly alter the counts of skeletal muscle-derived (SCG+), endothelial-derived (CD42A+), or platelet-derived (CD62E+) EV.

Based on other clinical studies, skeletal muscle EV release appears to be strongly influenced by mechanical contraction and energetic stress, such as physical exercise, rather than by endocrine stimulation alone, which might explain the absence of a detectable muscle EV response to rhGH [17,27].

Although skeletal muscle is a GH target tissue, many of its anabolic responses are mediated indirectly via tissue (i.e., local and non-peripherally liver-derived) IGF-1, and GH alone might be insufficient to trigger substantial EV release in the absence of mechanical stimuli, which is known to stimulate IGF-1 at the muscle level [28].

Similarly, endothelial and platelet EV release is primarily driven by shear stress, vascular injury, inflammation, or coagulation activation, none of which are typically induced by rhGH therapy in clinically stable pediatric patients [29].

The lack of change in CD42A+ and CD62E+ EVs further supports the cardiovascular safety profile of rhGH therapy, a topic extensively debated in the scientific community in previous decades, and suggests that rhGH, when appropriately administered, does not promote (abnormal) endothelial or platelet activation under these conditions [30].

We can, obviously, invoke other explanations, including the short duration of rhGH treatment and the relatively reduced sample size of our study, which might have impeded us from obtaining significant results.

### 4.4. Role of IGF-1 and Osteocalcin in EV Modulation

An important methodological and biological aspect of our preliminary study, worthy of being confirmed with a larger study population, is that significant changes in specific tissue-derived EV subpopulations emerged only after adjustment for IGF-1 and/or osteocalcin.

IGF-1 is the principal mediator of many GH effects and plays a central role in regulating cellular metabolism, growth, and survival, processes that are closely linked to vesicular trafficking and EV release [31].

Thus, statistically adjusting for IGF-1 likely allowed us to disentangle EV changes directly associated with GH signalling from those secondary to broader anabolic and metabolic effects of both GH and IGF-1 [32].

This argument seems to suggest that EV release during rhGH treatment is modulated not only by GH itself but also by its downstream endocrine mediators, e.g., IGF-1.

Osteocalcin, traditionally considered a bone formation marker, is now recognized as a hormone involved in glucose metabolism, insulin sensitivity, and energy expenditure [33].

The need to statistically adjust for osteocalcin indicates that bone-derived endocrine activity might indirectly influence circulating EV tissue-derived composition (at least or mainly, FABP+ Evs), highlighting bone as an active participant in systemic GH-dependent signalling networks.

### 4.5. Enhanced Vesiculogenic Response in Severe GHD

Children with more severe GHD seem to exhibit a more pronounced vesiculogenic response to rhGH, as indicated by interaction analyses with HV SDS, an important auxometric measure in clinical practice, for the rhGH-induced change in CD62E+ EVs.

This would suggest that chronic (or long-lasting peri-pubertal) GHD may prime target tissues for a hyper-response to GH replacement therapy, potentially through increased GH receptor expression or enhanced sensitivity to intracellular signalling [34].

Similar phenomena have been described in other endocrine deficiencies, where hormone replacement therapy elicits amplified cellular and molecular responses compared with physiological conditions [35].

Our previous argument is not supported by the results of GH peak to pharmacological tests (clonidine and arginine), which, in our interaction analyses, were not statistically significant. However, this finding should be considered with caution, given our relatively small study population and the well-known inter- and intra-individual variability in GH responses to pharmacological testing, which can create pitfalls in clinical practice when evaluating GHD severity [36].

### 4.6. Associations Between EVs and Clinical/Biochemical Parameters

The associations observed between specific tissue-derived EV subpopulations and auxological/auxometric or biochemical/metabolic parameters further support the functional relevance of EVs in GH action.

In particular, associations between CD14+ EVs and adiposity-related parameters seem to support the established role of immune-derived EVs in metabolic inflammation and lipid biochemistry [37].

Likewise, the associations between FABP+ EVs and insulin resistance, osteocalcin, and growth-related parameters seem to underscore the central role of adipose-tissue-derived EVs in coordinating metabolic adaptations and growth-related processes during rhGH therapy [26].

### 4.7. Limitations of the Study

Before closing, we should mention some limitations of our clinical study.

First of all, while a formal power calculation is somewhat challenging in pediatric population affected by a rare disease such as GHD, the results of the present clinical study should be considered only as preliminary, and enrolment of larger population of children with GHD is mandatory in future for confirming the effects of rhGH on EV release and for validating use of EVs as biomarker of rhGH effectiveness.

Second, even though the mean age (11.0 ± 2.7 years) suggests most children were pre- or early-pubertal, the statistical exclusion of age and pubertal stage as confounding factors should be cautiously considered due to the reduced sample size on one hand and the relevant role of sex hormone on GH responsiveness on the other hand [38].

Third, we have observed increased secretion of CD14+ and FABP+ EVs from immune system and adipose tissue cells, respectively. This is only a supposition, not supported by the results of our study, conducted in an in vivo model, i.e., children with GHD. Though it appears to be a plausible reason, further studies using in vitro models are mandatory.

### 4.8. Future Perspectives

Although our preliminary study provides the first in vivo evidence that rhGH therapy selectively modulates the tissue-derived composition of circulating EVs in children with GHD, several mechanistic and translational aspects warrant further investigation before EVs can be definitively proposed as biomarkers of treatment efficacy.

First, validation studies in larger, multicentre pediatric cohorts are required to confirm the reproducibility and robustness of the observed increases in CD14+ and FABP+ EV subpopulations. Such studies should incorporate longitudinal sampling at multiple time points (e.g., 1, 3, 6, and 12 months) to characterize the temporal kinetics of EV modulation in response to rhGH and to determine whether early EV changes anticipate auxological responses, including HV, osteocalcin, and IGF-1 increments. Establishing the predictive value of EV dynamics would be essential to support their clinical utility as early biomarkers of therapeutic responsiveness.

Second, mechanistic studies are needed to clarify whether the observed changes in EV subpopulations reflect increased vesicle biogenesis/release *per* cell, expansion of specific cellular compartments, or qualitative alterations in EV cargo. So, in vitro experiments using primary human monocytes/macrophages and adipocytes exposed to GH and/or IGF-1 under controlled conditions could dissect the relative contribution of GH receptor signalling pathways to EV biogenesis and release. Complementary analyses of EV cargo—including proteomics, transcriptomics, and miRNA profiling—would determine whether rhGH modifies not only EV quantity but also their functional molecular content, thereby influencing inter-organ communication.

Third, future studies should explore the relationship between EV profiles and clinical phenotypes beyond linear growth, particularly metabolic adaptations induced by rhGH. Given the associations observed between FABP+ EVs and insulin resistance–related parameters, prospective investigations integrating dynamic metabolic testing (e.g., oral glucose tolerance tests or clamp studies) could clarify whether specific EV signatures correlate with, or even precede, metabolic changes during therapy. Such data would be instrumental in evaluating whether EVs could serve as biomarkers not only of efficacy but also of metabolic risk stratification.

Fourth, comparative analyses, including control groups—such as healthy age-matched children or children with other causes of short stature (e.g., Prader–Willi syndrome, Turner syndrome, etc.)—would help determine the specificity of the EV signature identified in this study. This approach would clarify whether the modulation of immune- and adipose-derived EVs is a distinctive feature of GH replacement or a more general consequence of accelerated growth or endocrine activation.

Finally, standardization of pre-analytical and analytical procedures in accordance with current international guidelines for EV research will be crucial for enhancing reproducibility and facilitating cross-study comparisons. Integration of high-resolution imaging techniques and orthogonal platforms for EV characterization could further strengthen biological interpretation and accelerate translation into clinical practice.

Collectively, these future investigations will be necessary to confirm the mechanistic link between GH signalling and plasmatic EV release, to elucidate the functional role of EVs in GH-dependent inter-organ communication, and to establish circulating EV subpopulations as reliable, clinically meaningful biomarkers of rhGH treatment efficacy and disease severity in pediatric endocrinology.

## 5. Conclusions

Overall, rhGH therapy appears to selectively modulate the tissue-derived composition of circulating EVs in children with GHD, with immune- and adipose-tissue-derived EVs emerging as particularly responsive subpopulations. Our findings, though preliminary, suggest that plasma EVs might represent an adjunctive component of GH-dependent inter-organ communication and might serve as innovative biomarkers of treatment response and disease severity in pediatric endocrinology.

## Figures and Tables

**Table 1:** Demographic, biochemical, and clinical characteristics of participants (N = 10) in the study: comparison of pre- and post-rhGH treatment.

Characteristics	Baseline	Follow-Up	*p*-Value
Age, years	11.0 ± 2.7	11.6 ± 2.6	0.6961
Gender			
Male	5 (50%)	5 (50%)	—
Female	5 (50%)	5 (50%)	
Height, cm	133 [113; 139]	138 [119; 147]	0.2556
Height SDS	−2.5 ± 0.3	−2.2 ± 0.4	0.0712
Weight, kg	32.0 [19.1; 40.1]	35.4 [20.0; 45.1]	0.4814
Weight SDS	−2.2 [−2.4; −1.2]	−1.9 [−2.4; −0.8]	0.6288
BMI, kg/m^2^	17.0 [14.9; 20.3]	17.7 [14.7; 22.6]	0.9109
BMI SDS	−0.8 [−1.5; −0.1]	−0.9 [−1.5; 0.5]	1
FM, %	22.7 [15.4; 31.2]	21.9 [16.9; 28.5]	0.7941
FM, kg	5.8 [3.7; 12.5]	6.2 [4.5; 12.9]	0.8814
HV, cm/year	3.9 ± 1.4	8.7 ± 2.6	<0.0001
HV SDS	−2.8 [−3.8; −1.8]	1.7 [0.1; 4.1]	0.0014
GH peak to clonidine, mcg/L	4.6 ± 1.6	—	—
GH peak to arginine, mcg/L	4.4 ± 1.7	—	—
Glucose, mg/dL	81.8 ± 5.9	92.5 ± 6.1	0.0012
HbA1C, %	5.0 ± 0.2	5.3 ± 0.2	0.0226
Insulin, mU/L	5.0 ± 3.0	10.8 ± 4.7	0.0039
HOMA-IR	1.1 [0.7; 1.3]	2.4 [1.7; 2.6]	0.0114
TC, ng/dL	153 ± 21	177 ± 23	0.0258
HDL-C, mg/dL	52 ± 14	65 ± 19	0.0868
LDL-C, mg/dL	90 ± 16	103 ± 22	0.1727
TG, mg/dL	51 [44; 69]	55.5 [47; 87]	0.5279
RCP, mg/dL	0.0 [0.0; 0.3]	0.0 [0.0; 0.1]	0.8796
IGF-1, mcg/L	120.5 [102; 182]	341 [203; 510]	0.0076
Osteocalcin, mcg/L	96.5 [92; 105]	158 [135; 198]	0.0135

**Table 2:** Effects of 6-month rhGH treatment on EVs: linear mixed-effects regression analysis of paired data.

	Baseline			Follow-Up			Fold Change	95% CI		*p*-Value
	Geometric Mean	95% CI		Geometric Mean	95% CI					
**Total EVs**	6,001,801,235	4,589,226,886	7,849,169,143	6,627,058,891	5,462,452,571	8,039,961,715	1.10	1.19	1.02	0.5035
**CD62E+**	45,069	28,506	71,254	49,129.51787	34,996.6736	68,969.6843	1.09	1.23	0.97	0.7601
**CD14+**	25,320	19,682	32,572	37,602	28,261	50,031	1.49	1.44	1.54	0.0431
**SCG+**	99,974	59,324	168,480	131,775	75,355	230,438	1.32	1.27	1.37	0.5020
**CD42a+**	75,420	49,316	115,341	93,216	61,282	141,792	1.24	1.24	1.23	0.4919
**FABP+**	57,895	43,431	77,174	126,316	92,597	172,312	2.18	2.13	2.23	0.0045

Models were adjusted for IGF-1. For FABP+, models were additionally adjusted for osteocalcin. Geometric LS-means are shown, representing the estimated marginal means on the original scale after log-transformation, adjusted for covariates.

## Data Availability

The datasets used and/or analyzed in the present study will be uploaded on www.zenodo.org and will be available from the corresponding author upon a reasonable request.

## Supplementary Materials

The following supporting information can be downloaded at https://www.mdpi.com/article/10.3390/jcm15072528/s1. In particular: Figure S1: Association between EV concentrations means and rhGH treatment; Figure S2: *Spaghetti* plots showing individual trajectories of total and tissue-derived EVs from baseline (T0) to 6 months of rhGH treatment (T6); Table S1: Effect modification of height velocity (cm/year) on the changes in EVs between the two times: T6 (6 months of rhGH treatment) vs. T0 (baseline); Table S2: Effect modification of height velocity SDS on the changes in EVs between the two times: T6 (6 months of rhGH treatment) vs. T0 (baseline); Table S3: Effect modification of GH peak to clonidine on the changes in EVs between the two times: T6 (6 months of rhGH treatment) vs. T0 (baseline); Table S4: Effect modification of GH peak to arginine on the changes in EVs between the two times: T6 (6 months of rhGH treatment) vs. T0 (baseline); Table S5: Effect modification of height (SDS) on the changes in EVs between the two times: T6 (6 months of rhGH treatment) vs. T0 (baseline); Table S6: Effect modification of rhGH treatment on the association between Evs and clinical characteristics; Table S7: Association between Evs and clinical characteristics.

## Abbreviations

BA: body area; BMI, body mass index; BW: body weight; CI, confidence interval; CRP, C-reactive protein; EVs, extracellular vesicles; F, female; FFM, free fat mass; FM, fat mass; HBA1c, glycated hemoglobin; HDL-C, high-density lipoprotein cholesterol; HOMA-IR, homeostatic model assessment for insulin resistance; HV, height velocity; SDS, standard deviation score; IGF-1, insulin-like growth factor 1; LDL-C, low-density lipoprotein cholesterol; M, male; T-C, total cholesterol; TG, triglycerides.
